# Multilocus Sequence Typing Analysis of Carbapenem-Resistant *Acinetobacter baumannii* in a Chinese Burns Institute

**DOI:** 10.3389/fmicb.2016.01717

**Published:** 2016-11-09

**Authors:** Guangtao Huang, Supeng Yin, Yali Gong, Xia Zhao, Lingyun Zou, Bei Jiang, Zhiwei Dong, Yu Chen, Jing Chen, Shouguang Jin, Zhiqiang Yuan, Yizhi Peng

**Affiliations:** ^1^State Key Laboratory of Trauma, Burns and Combined Injury, Institute of Burn Research, Southwest Hospital, Third Military Medical UniversityChongqing, China; ^2^Department of Microbiology, Bioinformatic Center, College of Basic Medical Sciences, Third Military Medical UniversityChongqing, China; ^3^Department of Molecular Genetics and Microbiology, College of Medicine, University of Florida, GainesvilleFL, USA

**Keywords:** *A. baumannii*, nosocomial infection, multi locus sequence typing (MLST), β-lactamases, clonal complex (CC)

## Abstract

*Acinetobacter baumannii* is a leading pathogen responsible for nosocomial infections. The emergence of carbapenem-resistant *A. baumannii* (CRAB) has left few effective antibiotics for clinicians to use. To investigate the temporal evolutionary relationships among CRAB strains, we collected 248 CRAB isolates from a Chinese burns institute over 3 years. The prevalence of the *OXA-23* gene was detected by polymerase chain reaction. Multilocus sequence typing was used to type the CRAB strains and eBURST was used to analyze their evolutionary relationships. Wound surfaces (41%), sputa (24%), catheters (15%), and bloods (14%) were the four dominant isolation sources. Except for minocycline (33.5%) and sulbactam/cefoperazone (74.6%), these CRAB strains showed high resistance rates (>90%) to 16 tested antibiotics. The 248 isolates fall into 26 sequence types (STs), including nine known STs and 17 unknown STs. The majority (230/248) of these isolates belong to clonal complex 92 (CC92), including eight isolates belonging to seven unreported STs. A new CC containing 11 isolates grouped into four new STs was identified. The *OXA-23* gene was detected at high prevalence among the CRAB isolates and the prevalence rate among the various STs differed. The majority of the isolates displayed a close evolutionary relationship, suggesting that serious nosocomial spreading and nosocomial infections of CRAB have occurred in the burn unit. In conclusion, the main CC for CRAB in this Chinese burn unit remained unchanged during the 3-year study period, and a new CC was identified. CC92 was the dominant complex, and more attention should be directed toward monitoring the new CC we have identified herein.

## Introduction

Gram-negative, non-fermentative *Acinetobacter baumannii* has emerged as one of the most troublesome nosocomial infectious pathogens (Fournier et al., 2006; Dijkshoorn et al., 2007; Peleg et al., 2008). To highlight the serious situation regarding antibiotic-resistant pathogens, the concept of ESKAPE pathogens has recently been introduced, and the pathogens belonging to it include *Enterococcus faecium, Staphylococcus aureus, Klebsiella pneumoniae, A. baumannii, Pseudomonas aeruginosa*, and *Enterobacteriaceae* (Rice, 2010; Woksepp et al., 2014). These organisms have become a major concern for scientists and policy makers alike in many countries.

Although imipenem (IMP) and meropenem (MEM) are still the first choice treatments for multidrug-resistant *A. baumannii* infections in the clinic, CRAB has been increasingly reported across the world (Hagihara et al., 2014; Hammerum et al., 2015). CRAB has also drawn public concern because of the limited number of effective clinical antibiotics remaining in use. Several genes and mechanisms underlie carbapenem-resistance, and these include β-lactamases, mutations in bacterial outer membrane proteins, and multidrug efflux pumps. β-lactamases, especially OXA-type carbapenemases (class D β-lactamases), are common determinants of carbapenem resistance (Peleg et al., 2008; Hammerum et al., 2015). Currently, at least five subgroups of β-lactam resistance genes have been identified, and these genes share certain similarities in their DNA sequences. Among the class D β-lactam resistance genes, *OXA-23* is the most widespread resistance gene in *A. baumannii* (Farshadzadeh et al., 2015; Jia et al., 2015; Zowawi et al., 2015).

Multilocus sequence typing is an effective method to accurately characterize pathogens at molecular level, and is based on fragments of seven housekeeping genes (Bartual et al., 2005). It has become a popular typing method and has achieved notable success in comparing epidemiological investigations across different geographical areas. Previous investigations have shown that molecular types of *A. baumannii* vary in different regions (Ruan et al., 2013; Farshadzadeh et al., 2015). In the present study, we collected 248 CRAB strains from January 2012 to December 2014 and using MLST analysis found that the majority of these CRABs belonged to the CC known as CC92. Although the sequence type (ST) 368 has maintained the dominant position for 3 years, the ST population has changed year by year. Further phylogenetic analysis showed that the majority of the 248 isolates fell into cluster 1. Therefore, it is necessary to implement better control of nosocomial CRAB infections, and to survey the prevalence of such infections in the burns unit where this study was conducted.

## Materials and Methods

### Ethics Statement

The study was approved by the Committee of the First Affiliated Hospital of Third Military Medical University, China. No written informed consent was required because we received anonymized isolate samples with all the personal information removed. Patient names or any other personal information was not present in the data.

### Collection and Identification of *A. baumannii* Isolates

A total of 248 CRAB isolates were collected from the Burns Institute at Southwest Hospital in southwest China. All the isolates were phenotypically identified to genus level by phenotypic methods (API 20 NE system, Biomerieux). *A. baumannii* species genotypic identification was performed by PCR detection of the *OXA-51* gene (Turton et al., 2006; Zander et al., 2013). Isolates lacking the *OXA-51* gene were investigated further by sequence analysis of the 16S rRNA gene (Huang et al., 2013, 2014).

### Antimicrobial Susceptibility Testing

For antimicrobial susceptibility testing, 19 commonly used clinical antibiotics were analyzed using the K-B method. The 19 antibiotics tested were amikacin (AMK), ampicillin/sulbactam (SAM), polymyxin B (POL), sulfamethoxazole/trimethoprim (SXT), ciprofloxacin (CIP), meropenem (MEM), minocycline (MNO), netilmicin (NET), gentamicin (GEN), tetracycline (TCY), ceftazidime (CAZ), cefepime (FEP), sulbactam/cefoperazone (CSL), cefotaxime (CTX), tobramycin (TOB), imipenem (IPM), levofloxacin (LVX), piperacillin (PIP), and piperacillin/tazobactam (TZP). The results were interpreted according to the Clinical and Laboratory Standards Institute (CLSI) criteria (2015).

### Detection and Characterization of the *OXA-23* Gene

Genomic DNA from the isolates was extracted with a Rapid Bacterial Genomic DNA Isolation Kit (Sangon, China) following the manufacturer’s protocol. *OXA-23* primers (Mugnier et al., 2010) and *OXA-51* primers (Turton et al., 2006) were synthesized at the Beijing Genome Institute. The PCR conditions used were as follows: denaturation at 95°C for 5 min, followed by 30 cycles of 95°C for 30 s, annealing for 30 s, and a 72°C extension for 90 s, and 72°C for 10 min. PCR products were analyzed by agarose gel electrophoresis. The theoretical sizes of the *OXA-23* and *OXA-51* PCR products are 1067 and 353 bp, respectively.

### Multi Locus Sequence Typing

Multi locus sequence typing is a molecular epidemiology method used widely in recent years for distinguishing molecular subtypes of bacteria. Compared with pulsed-field gel electrophoresis, MLST can be used to compare isolates from different countries and geographical areas more conveniently. After DNA extraction, seven *A. baumannii* chromosomal genes recommended by the MLST database website^1^ were amplified and sequenced using an ABI 3700 DNA sequencer. The sequencing results for each gene were compared with the known sequences in the database to obtain the locus number. STs can be identified by seven locus numbers. New types will be defined if seven loci do not match known types.

### eBURST and Phylogenetic Analysis

eBURST version 3.0^2^ was used to analyze the MLST data to determine the evolutionary relationships among the isolates (Feil et al., 2004; Francisco et al., 2009). All the reported MLST data were downloaded from the PubMLST database^3^. The default definition (sharing six of seven total alleles) was used to find the CC for the groups. Clustal X2 was used for the multi-sequence alignments and the alignment results were also used to construct a phylogenetic tree using the maximum parsimony method within PhyML(Guindon et al., 2010).

### Statistics Analysis

SPSS version 18.0 (SPSS, Inc., Chicago, IL, USA) was used for the statistical analysis. The Pearson Chi-Squared test was performed to compare the differences in the *OXA-23* prevalence rates between different STs and different years. Differences were considered significant when *p* < 0.05.

## Results

### Collection and Characterization of *A. baumannii* Strains

The Burns Institute of Southwest Hospital is a burn center with 150 beds that takes patients from southwest China mainly, including the provinces of Chongqing, Sichuan, Guizhou, and Yunnan. To conduct this study, we collected and analyzed CRAB isolates from the biological sample library at the Burns Institute. A total of 248 CRAB isolates were obtained over 3 years (January 2012 to December 2014). The number of isolates in 2012, 2013, and 2014 was 66, 93, and 89, respectively. The *OXA-51* gene, which is reported to be an *A. baumannii*-specific gene, was found in 242 of the 248 isolates. The remaining six *OXA-51*-negative isolates were confirmed to be *A. baumannii* by 16S rRNA gene sequencing.

**FIGURE 1:**
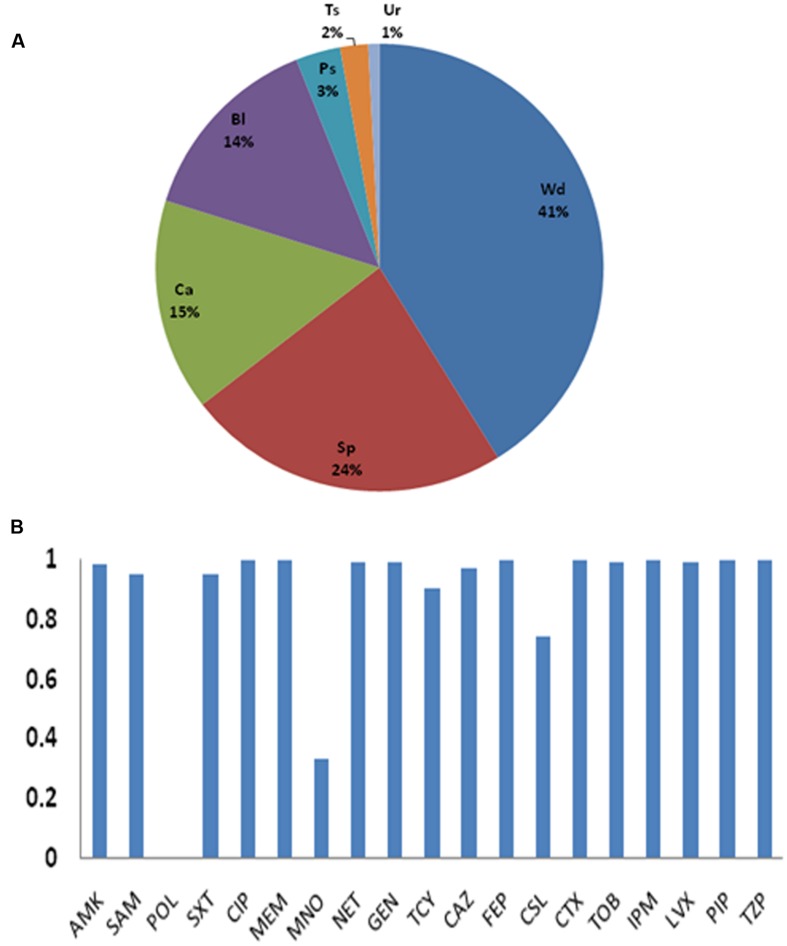
**(A)** Sources of the 248 strains. Wd (wound surface), Sp (sputa), Ca (catheter), Bl (bloodstream), Ps (purulent secretion), Ts (tissue), and Ur (urine). **(B)** Antibiotic resistance rates. AMK, amikacin; SAM, ampicillin/sulbactam; POL, Polymyxin B; SXT, sulfamethoxazole/trimethoprim; CIP, ciprofloxacin; MEM, meropenem; MNO, minocycline; NET, netilmicin; GEN, gentamicin; TCY, tetracycline; CAZ, ceftazidime; FEP, cefepime; CSL, sulbactam/cefoperazone; CT, cefotaxime; TOB, tobramycin; IPM, imipenem; LVX, levofloxacin; PIP, piperacillin; TZP, piperacillin/tazobactam.

As shown in **Figure 1A**, the isolates were from wound surfaces (41%), sputa (24%), catheters (15%), bloods (14%), purulent secretions (3%), tissues (2%), and urines (1%). Thus, wound surfaces, sputa, catheters, and bloods were the dominant sources, and these covered 94% of all the isolates.

### Antimicrobial Susceptibility

Most of the CRAB isolated in this center showed the multi-drug resistant phenotype (**Figure 1B** and Supplementary Material). Except for MEM and IPM, all the isolates were also resistant to CIP, FEP, CTX, PIP, and TZP. Although, no resistance was found against polymyxin B, its use is very limited in clinical work due to its toxicity. The resistance rates for MNO and CSL, which are the only antibiotics left for treating CRAB infections, were 33.5 and 74.6%.

### Multilocus Sequence Typing Profile Analysis

Nine known STs and 17 unknown STs were found in this study. The nine known STs together with the number typed (in brackets) are as follows: ST136 (2), ST191 (29), ST195 (31), ST208 (21), ST229 (1), ST368 (103), ST369 (29), ST457 (1), and ST381 (7). The profiles of the newly identified ST types are listed in **Table 1**. ST368 is the dominant ST type (41.1%) in the isolates. Twenty-five isolates belong to unreported ST types. Additionally, two isolates, six isolates, and three isolates belong to new8, new9, and new14, respectively. All other new STs contained only one isolate.

**Table 1:** Allelic profiles of the new STs found in this study.

STs	*gltA*	*gyrB*	*gdhB*	*recA*	*cpn60*	*gpi*	*rpoD*
new1	1	81	3	2	46	16	3
new2	1	34	80	28	1	16	30
new3	49	3	3	19	28	96	69
new4	1	64	3	1	23	106	26
new5	1	34	80	28	1	106	45
new6	1	3	3	2	4	106	3
new7	10	90	37	6	2	106	5
new8	1	3	3	2	46	140	3
new9	10	12	37	6	4	140	5
new10	10	12	37	6	2	140	5
new11	0^∗^	3	3	2	2	140	3
new12	1	81	3	2	2	140	3
new13	10	3	37	6	4	140	5
new14	10	12	37	2	4	140	5
new15	1	3	37	67	2	140	3
new16	1	3	3	6	2	140	3
new17	1	81	3	2	2	0^∗^	3


*0^∗^ means that this gene sequence does not match any previously reported sequences.*

**FIGURE 2:**
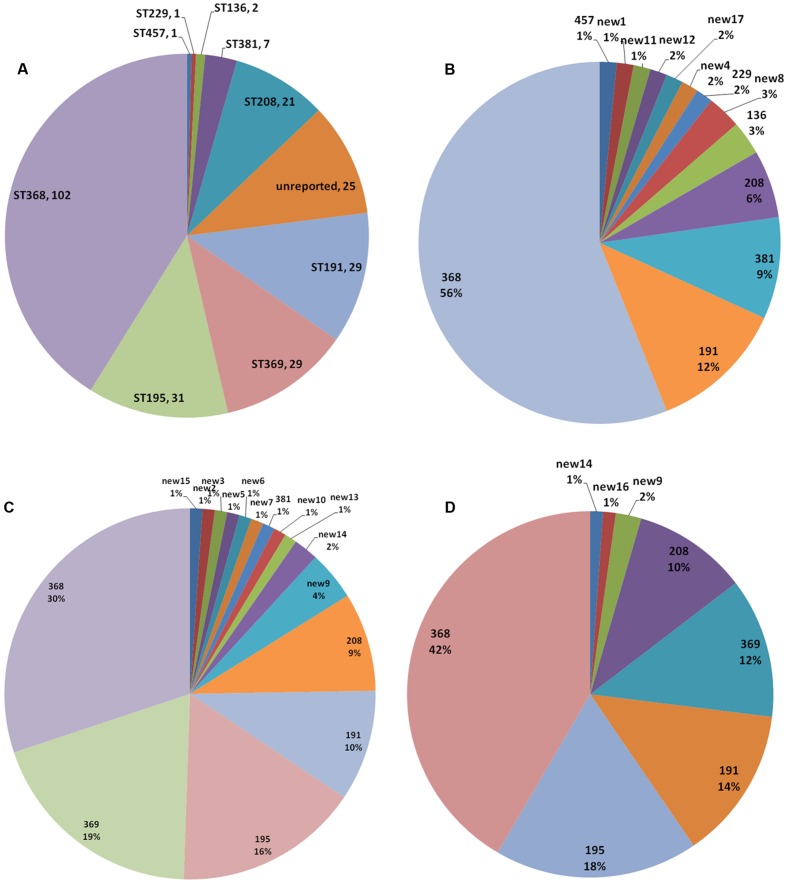
**Multi locus sequence typing population analysis.** STs in the 248 isolates **(A)** and the STs identified in 2012 **(B)**, 2013 **(C)**, and 2014 **(D)**.

Although, the dominant ST (ST368) remained unchanged during the 3-year study period (**Figure 2**), the ratio decreased from 56% in 2012 to 30% in 2013, and rose to 42% in 2014. None of 66 isolates from 2012 belonged to ST369. However, 18 strains (19%) of ST369 were isolated in 2013. The number decreased to 11 (12%) in 2013. As with ST369, ST195 strains also emerged suddenly in 2013, as many as 15 strains (16%). ST195 even rose to 18% in 2014. The ST population changed year by year, with some minor types diminishing or switching to another minor type (**Figures 2B–D**). For example, the number of ST381 isolates decreased from 6 in 2012 to 1 in 2013 and no ST381 isolate was found in 2014.

### eBURST Analysis

**FIGURE 3:**
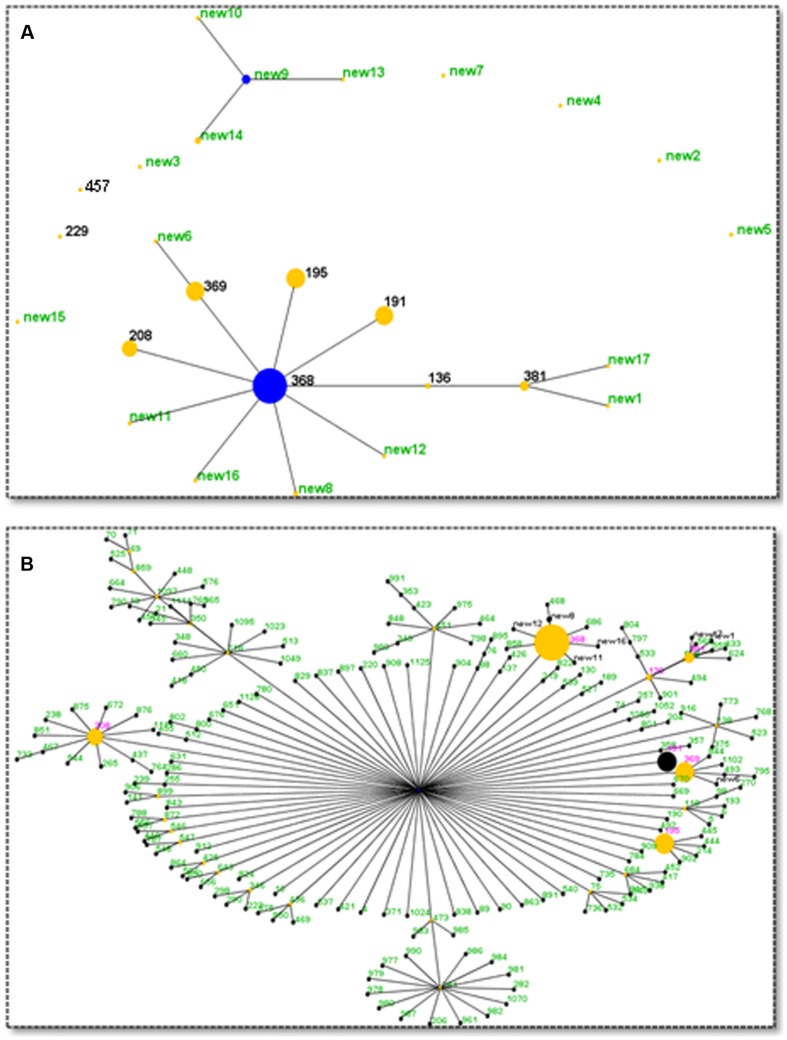
**eBURST analysis results.** **(A)** Relationships among the STs found in this study. Two clonal complexes were identified. Blue dot denotes the founder and yellow dots denote the sub-founder. The size of the dot indicates the number of isolates. **(B)** Current members (STs) of CC92. Seven CC92 STs identified in this study are shown in red, and seven new CC92 STs are shown in black. Other previously reported CC92 STs are shown in green.

eBURST is an algorithm that can divide MLST data into groups and CCs, and can also infer patterns of evolutionary descent (Feil et al., 2004). With the default definition (sharing six of seven alleles), a CC is a set of STs (at least three) that are believed to be descended from the same founding genotype. As reported previously, many CRAB isolates all over the world belong to CC92, the most common CC. Except for ST229, all the other seven known STs (named in pink in **Figure 3**) belong to CC92, which covers 89.5% (222/248) of all isolates. Additionally, seven newly found STs, which contain 3.2% (8/248) of all isolates, also belong to CC929 (**Figure 3**). Until now, 211 members were identified as belonging to CC92, including the seven new STs (named in black in **Figure 3B**). ST208, ST368, ST369, and ST195 were also identified as sub-founders.

A new CC containing four new STs (new9, new10, new13, and new14) was found in this investigation (**Figure 3A**). This new CC contains 11 isolates, with six isolates of new9, one isolate each for new10 and new13, and three isolates of new14. Seven of them were isolated from wood surfaces and two from blood. The two remaining were from sputa and catheter samples. Two isolates were from 2012 and nine strains from 2013. In summary, 230 isolates belong to CC92 and 11 isolates belong to the new CC. No genetic relationship was found among the remaining seven isolates.

### Detection of the *OXA-23* Gene

**FIGURE 4:**
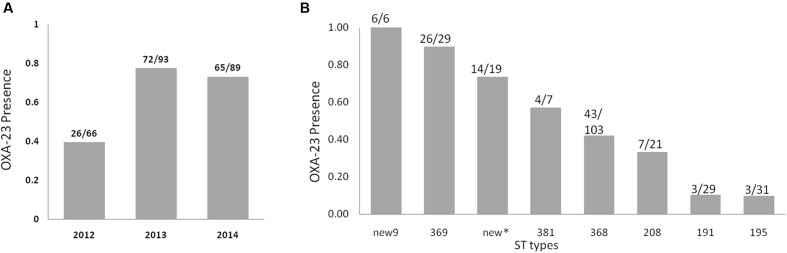
***OXA-23* gene prevalence among *Acinetobacter baumannii* isolates in each year** **(A)** and in different STs **(B)**. New^∗^ represent all the new STs except new9.

*OXA-23* is among the most widely spread drug-resistant genes in *A. baumannii*. In this investigation, we found the prevalence of the *OXA-23* gene was 65.7% (163/248). One investigation over 8 years in south China reported that the prevalence of *OXA-23* was approximately 51.5% in CRAB isolates (Wu et al., 2015). Another study recently reported a somewhat lower prevalence of *OXA-23* (40%) in Iran (Bahador et al., 2015). The prevalence of *OXA-23* increased significantly from 39% in 2012 to 77% in 2013 and 73% in 2014 (**Figure 4A**, *p*-value < 0.001, chi-square value = 28.069). The *OXA-23* gene prevalence altered significantly among the different STs (**Figure 4B**, *p*-value < 0.001, chi-square value = 68.996). For ST195 and ST191, only 10% of these isolates carried *OXA-23*; however, 26 of 29 ST369 strains carried it. For new9, all six isolates carried *OXA-23*. The prevalence of *OXA-23* in new9, ST369, and the other new types, namely, ST381, ST368, ST208, ST191 and ST195, was 100, 90, 74, 57, 42, 33, 10, and 10%, respectively.

### Phylogenetic Analysis

**FIGURE 5:**
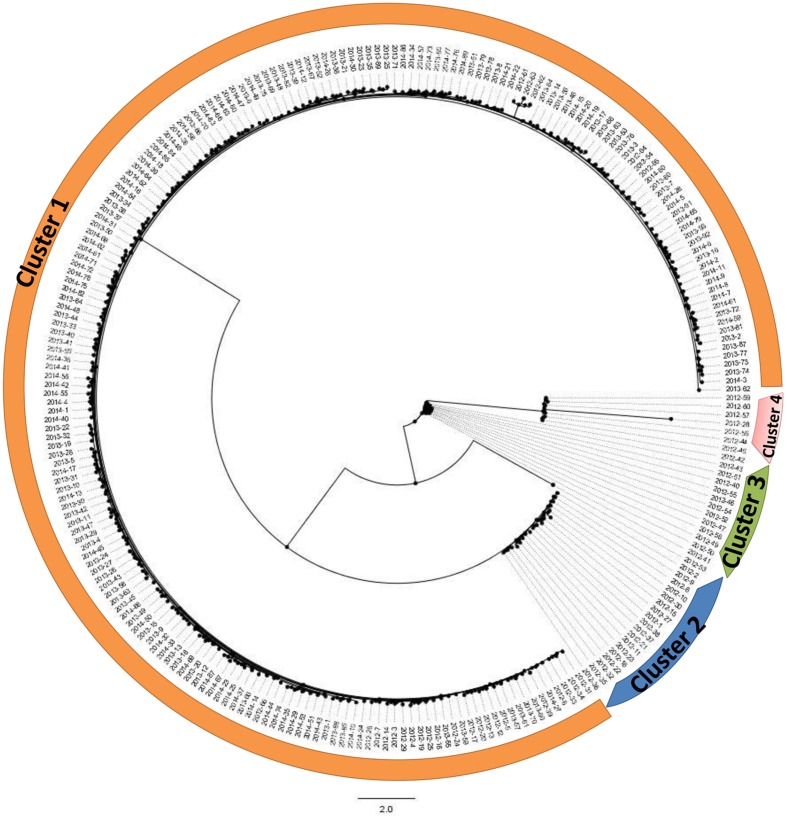
**Phylogenetic analysis based on concatenated sequences of seven loci.** All 248 isolates are mainly divided into four clusters.

These isolates were mainly divided into four clusters (**Figure 5**). Cluster 1 is the majority cluster, containing 209 isolates. A total of 18, 13, and 7 isolates were grouped into cluster 2, cluster 3, and cluster 4. The strains of these three clusters were all isolated from 2012. The strains isolated from 2013 and 2014 were mainly grouped into cluster 1.

## Discussion

*Acinetobacter baumannii* is a leading pathogen responsible for hospital-acquired infections. Its ability to rapidly develop antibiotic resistance means that it has become a great concern to scientists and clinicians worldwide (Dijkshoorn et al., 2007; Peleg et al., 2008). CRAB was first described around the year 2000 and its prevalence has risen quickly among Gram-negative hospital isolates. The prevalence of this bacterium is even higher among burn intensive care units (Yali et al., 2014).

As was found in a previous study, CC92 was the dominant CC in the Chinese mainland (Ruan et al., 2013). In fact, CC92 is also the dominant complex across the world. The members of this CC keep growing in number (Ruan et al., 2013). Although, a series of measures have been taken to control *A. baumannii* infections, the infections caused by CC92 CRAB are still reported occasionally in clinical work. According to our eBURST analysis, the majority of the 248 isolates displayed a relatively close evolutionary relationship in the phylogenetic tree. These results indicate the wide clonal spreading of CRAB in the Chinese burn unit.

All known STs have been reported in China, where ST92 was the main ST before 2010 (Ruan et al., 2013), and ST195 together with ST208 are currently the main STs (Zhou et al., 2015; Qu et al., 2016). In the present study, ST195 and ST208 were moderately common STs and ST368 was the dominant type. Although, the majority of isolates over the 3-year study were CC92, the CC92 populations were observed to change temporally. Two moderately common STs (ST369 and ST195) emerged after 2013 (**Figure 2**). No ST369 isolates were found in 2012. However, 18 and 11 strains were isolated in 2013 and 2014, respectively. The ST369 isolates showed a high *OXA-23* carriage rate (90%). However, the *OXA-23* carriage rate in ST195 isolates was low (10%). Some minor STs emerged only once, and most of these were new STs (Supplementary Material). However, new9 contains six isolates (four in 2013 and two in 2014), and all of them carry the *OXA-23* gene.

*OXA-23* encodes the hydrolase enzyme of β-lactam, which has been reported in different countries (Ruan et al., 2013; El-Shazly et al., 2015; Quinones et al., 2015). In this study, the prevalence rate for *OXA-23* was, at 67%, high. In fact, the OXA-23 carriage rate in China is relatively high. Some investigations have shown that the prevalence reached over 90% in coastal areas (Ji et al., 2014; Zhou et al., 2015). Additionally, the prevalence of the *OXA-23* gene varies among isolates with different STs. The prevalence also differs between CC92 and the new CC. For the CC92 strains, 100 of the 222 isolates carried *OXA-23*, and all 11 CRAB isolates from the new CC carried it. One limitation of the present study is that only the *OXA-23* gene was detected, whereas many other β-lactam-resistance genes have been reported. For the *OXA-23*-negative isolates from this study, it is possible that they carry other β-lactam-resistance genes or other drug-resistance determinants may be present in them, such as outer membrane protein mutations and multidrug efflux pumps.

To conclude, we have collected 248 CRAB strains over a 3-year period from a burn unit and found that *OXA-23* was highly prevalent among the isolates. Many unreported STs were identified, some of which belonging to CC92. A new CC was identified that contained 11 isolates belonging to four new STs. Although the new CC was in the minority, the isolates within it may give rise to future disease outbreaks, therefore, monitoring this CC is needed in this burn unit.

## Author Contributions

YP, ZY, and GH conceived and designed this study. GH, YG, SY, and BJ carried out the experiments. XZ and LZ constructed the phylogenetic tree. GH, YC, ZD, JC, ZY, and YP analyzed the data. GH and SJ drafted the manuscript. All authors have read and approved the final manuscript.

## Conflict of Interest Statement

The authors declare that the research was conducted in the absence of any commercial or financial relationships that could be construed as a potential conflict of interest.

The reviewer YS and handling Editor declared their shared affiliation and the handling Editor states that the process nevertheless met the standards of a fair and objective review.

## Footnotes

1 http://pubmlst.org/abaumannii/

2 http://eburst.mlst.net/

3 http://pubmlst.org/

## Abbreviations

CC: clonal complex
CLSI: Clinical and Laboratory Standards Institute
CRAB: carbapenem-resistant A. baumannii
MLST: multilocus sequence typing
